# Risk factors and prognostic indicators for progressive fibrosing interstitial lung disease: a deep learning-based CT quantification approach

**DOI:** 10.1007/s00330-025-11714-x

**Published:** 2025-06-17

**Authors:** Kanghwi Lee, Jong Hyuk Lee, Seok Young Koh, Hyungin Park, Jin Mo Goo

**Affiliations:** 1 Department of Radiology, Seoul National University Hospital, Seoul, Korea; 2 Department of Radiology, Seoul National University College of Medicine, Seoul, Korea; 3 Department of Radiology, Inje University College of Medicine, Haeundae Paik Hospital, Busan, Korea; 4 Institute of Radiation Medicine, Seoul National University Medical Research Center, Seoul, Korea; 5 Cancer Research Institute, Seoul National University, Seoul, Korea

**Keywords:** Interstitial lung disease, Progressive fibrosing interstitial lung disease, Quantitative computed tomography, Fibrosis extent, Chest radiology

## Abstract

**Objectives:**

To investigate the value of deep learning-based quantitative CT (QCT) in predicting progressive fibrosing interstitial lung disease (PF-ILD) and assessing prognosis.

**Materials and methods:**

This single-center retrospective study included ILD patients with CT examinations between January 2015 and June 2021. Each ILD finding (ground-glass opacity (GGO), reticular opacity (RO), honeycombing) and fibrosis (sum of RO and honeycombing) was quantified from baseline and follow-up CTs. Logistic regression was performed to identify predictors of PF-ILD, defined as radiologic progression along with forced vital capacity (FVC) decline ≥ 5% predicted. Cox proportional hazard regression was used to assess mortality. The added value of incorporating QCT into FVC was evaluated using C-index.

**Results:**

Among 465 ILD patients (median age [IQR], 65 [58–71] years; 238 men), 148 had PF-ILD. After adjusting for clinico-radiological variables, baseline RO (OR: 1.096, 95% CI: 1.042, 1.152, *p* < 0.001) and fibrosis extent (OR: 1.035, 95% CI: 1.004, 1.067, *p* = 0.025) were PF-ILD predictors. Baseline RO (HR: 1.063, 95% CI: 1.013, 1.115, *p* = 0.013), honeycombing (HR: 1.074, 95% CI: 1.034, 1.116, *p* < 0.001), and fibrosis extent (HR: 1.067, 95% CI: 1.043, 1.093, *p* < 0.001) predicted poor prognosis. The Cox models combining baseline percent predicted FVC with QCT (each ILD finding, C-index: 0.714, 95% CI: 0.660, 0.764; fibrosis, C-index: 0.703, 95% CI: 0.649, 0.752; both *p*-values < 0.001) outperformed the model without QCT (C-index: 0.545, 95% CI: 0.500, 0.599).

**Conclusion:**

Deep learning-based QCT for ILD findings is useful for predicting PF-ILD and its prognosis.

**Key Points:**

***Question*** *Does deep learning-based CT quantification of interstitial lung disease (ILD) findings have value in predicting progressive fibrosing ILD (PF-ILD) and improving prognostication?*

***Findings*** *Deep learning-based CT quantification of baseline reticular opacity and fibrosis predicted the development of PF-ILD. In addition, CT quantification demonstrated value in predicting all-cause mortality.*

***Clinical relevance*** *Deep learning-based CT quantification of ILD findings is useful for predicting PF-ILD and its prognosis. Identifying patients at high risk of PF-ILD through CT quantification enables closer monitoring and earlier treatment initiation, which may lead to improved clinical outcomes.*

**Graphical Abstract:**

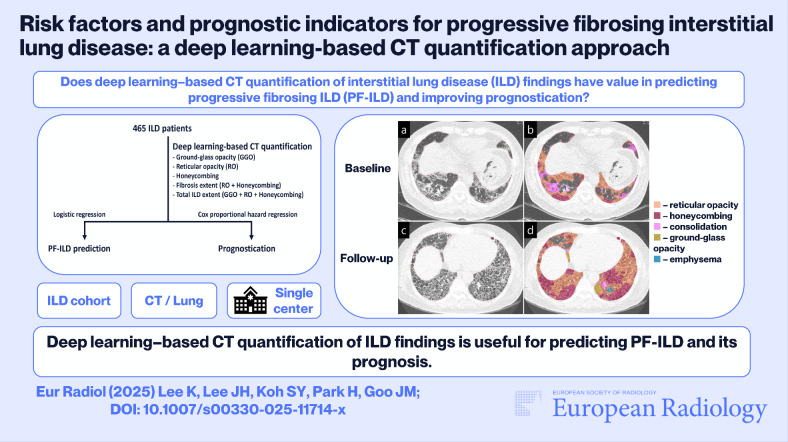

## Introduction

Progressive fibrosing interstitial lung disease (PF-ILD) is a clinical phenotype marked by increased fibrosis visible on CT scans, declining lung function, and worsening symptoms, which ultimately lead to early mortality [1–4]. The INBUILD study, which evaluated the effectiveness of a tyrosine-kinase inhibitor (nintedanib) in patients with PF-ILD, defines this condition as follows [1, 5]: despite standard treatment, PF-ILD is characterized by (1) a relative decline in forced vital capacity (FVC) of ≥ 10% of the predicted value, (2) a relative decline in FVC of ≥ 5% along with increased fibrosis on CT or worsening symptoms, or (3) worsening symptoms accompanied by increased fibrosis on CT. Beyond idiopathic pulmonary fibrosis (IPF), which is synonymous with progressive fibrosis, a subset of non-IPF ILD patients also exhibit features of progressive fibrosis. This subset includes those with idiopathic nonspecific interstitial pneumonia (NSIP), connective tissue disease-associated ILD (CTD-ILD), and fibrotic hypersensitivity pneumonitis (HP) [5–7]. Regardless of the ILD subtype, patients with non-IPF PF-ILD experience a short-term decline in FVC and early mortality rates comparable to those observed in patients with IPF [1, 5, 6, 8].

Chest CT is essential for diagnosing, monitoring, and predicting outcomes in ILD [1, 5, 9, 10]. Studies have extensively examined radiologic risk factors for PF-ILD in ILD patients, including the usual interstitial pneumonia (UIP) CT pattern and extensive traction bronchiectasis observed on CT scans [11–15]. However, visual assessments of CT images often lead to significant inter- and intra-reader variability, such as moderate agreement for honeycombing and fair to moderate agreement for classifying ILD categories, underscoring the importance of quantitative CT (QCT) analysis [16–19]. Indeed, various QCT methods for assessing ILD extent have proven to be essential tools for evaluating disease progression and predicting changes in lung function [20–22]. Notably, advancements in deep learning-based QCT have demonstrated considerable promise for classifying ILD subtypes and predicting outcomes in PF-ILD patients [23, 24]. This study explores the utility of deep learning-based QCT in identifying risk factors for PF-ILD.

## Materials and methods

This retrospective study was approved by the Institutional Review Board (IRB) of Seoul National University Hospital, and the requirement for written informed consent was waived (IRB No. H-2305-086-1431).

### Study sample

This retrospective cohort study was performed at a tertiary referral hospital in South Korea. All CT scans between January 2015 and June 2021 with keywords describing ILD in radiological reports (e.g., “reticular opacity,” “reticulation,” “traction bronchiectasis,” “honeycombing,” “interstitial fibrosis,” “interstitial lung disease,” “ILD,” “idiopathic pulmonary fibrosis,” “IPF,” “usual interstitial pneumonia,” “UIP,” “nonspecific interstitial pneumonia,” “NSIP,” “hypersensitivity pneumonitis,” “HP,” “interstitial lung abnormality,” or “ILA”). The search yielded 2864 patients.

**Fig. 1:**
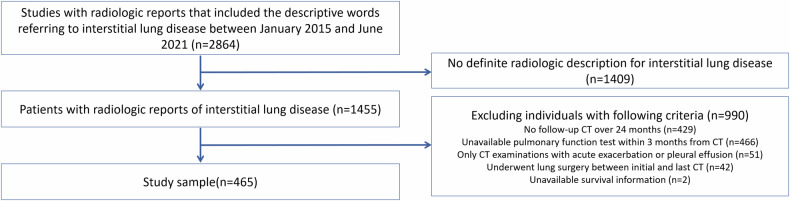
Flowchart for the selection process of the study population

The exclusion criteria were as follows: patients whose radiologic reports did not indicate ILD (*n* = 1409); those with CT examination intervals of less than 24 months between baseline and final follow-up scans during the study period (*n* = 429), as PF-ILD is defined by a decline in PFT results or an increase in fibrosis on CT over 24 months [7]; and those without available PFT results within 3 months of each CT scan time point (*n* = 466). A thoracic radiologist (J.H.L., with 11 years of experience in ILD imaging) and a board-certified radiologist (S.Y.K., with 4 years of experience in ILD imaging) reviewed the remaining CT images. They excluded additional patients whose CT images showed either an acute exacerbation of ILD or a significant amount of pleural effusion that obscured the ILD findings in the lung parenchyma (*n* = 51), and those who had undergone lung surgery between the baseline and follow-up scans (*n* = 42). Patients for whom survival information was not available were also excluded (*n* = 2) (Fig. 1). The study population overlaps with a previous publication by Koh et al [24], with additional 3 patients excluded due to acute exacerbation (*n* = 1) and unavailable survival information (*n* = 2).

### Visual assessment for ILD

The radiologic ILD pattern on baseline CT was visually categorized by a radiology resident (K.L., with 3 years of experience in ILD imaging) and a thoracic radiologist (J.H.L.) according to the recent IPF guidelines [25, 26]. The patterns were grouped as follows: (1) UIP pattern, (2) probable UIP pattern, and (3) indeterminate for UIP or an alternative diagnosis.

Visual fibrosis progression on CT was independently assessed by two thoracic radiologists (J.H.L. and H.I.P., with 6 years of experience in ILD imaging). They reviewed baseline and follow-up images side-by-side to determine ILD progression as either progression or stability, based on the following guideline criteria [26]: (1) increased extent or severity of traction bronchiectasis and bronchiolectasis; (2) new ground-glass opacity with traction bronchiectasis; (3) new or increased extent or coarseness of reticular opacity; (4) new or increased honeycombing; (5) increased lung volume loss. If their evaluations were in agreement, that consensus was used. In cases of disagreement, a senior thoracic radiologist, J.M.G., with 32 years of experience in ILD imaging, made the final determination on visual progression.

Since all patients had at least a 24-month interval from the baseline CT scan, a proportion of them also underwent intermediate 1-year follow-up CT scans. To investigate the implications of 1-year changes in QCT, we collected these available follow-up CT scans performed between 9 and 15 months after baseline.

### QCT analyses

A commercially available deep learning-based texture analysis software (AVIEW Lung Texture version 1.1.43.7, Coreline Soft) was utilized to segment ILD findings, including emphysema, consolidation, ground-glass opacity (GGO), reticular opacity (RO), and honeycombing. Each finding was automatically segmented and quantified as a percentage of the total lung volume.

Fibrosis extent was defined as the sum of RO and honeycombing, and total ILD extent was defined as the sum of GGO, RO, and honeycombing [1, 27, 28]. A thoracic radiologist (J.H.L.) applied this software to all baseline and follow-up CT images and confirmed the completeness of segmentation without additional manual modifications.

Details of CT image acquisition are provided in the Supplementary Text.

### Clinical data

The following clinical data were acquired from electronic medical records: age, sex, body mass index (BMI), ILD subtypes, and smoking status when available (ever vs. never). Additionally, FVC (volume, L) and predicted FVC (%) results, taken within 3 months of baseline and follow-up CT scans, were collected according to the guidelines of the American Thoracic Society [29]. This study utilized the absolute decline in percent predicted FVC between baseline and follow-up PFTs, in accordance with recent PFT guidelines [26].

Survival status and date of death were obtained from a database maintained by the Ministry of the Interior and Safety, Korea. Overall survival was defined as the period from the baseline CT scan until death from any cause. Survival time was censored as of September 28th, 2022. For patients who died, the censoring time was recorded as the date of death.

### Statistical analyses

Baseline characteristics, PFT results, and ILD CT quantification results are presented as means with standard deviations or medians with interquartile ranges (IQRs), depending on the results of normality testing. From the three definitions of PF-ILD provided by the INBUILD trial, we were unable to evaluate patients’ symptoms worsening due to the retrospective design of our study [1, 5]. Instead, PF-ILD was defined based on visual fibrosis progression on CT and an absolute decline in percent predicted FVC of ≥ 5%. To assess the predictive value of QCT for PF-ILD and all-cause mortality, we conducted multivariable logistic and Cox proportional hazard regression analyses. These analyses included each ILD finding (GGO, RO, honeycombing) in multivariable model 1, fibrosis extent in multivariable model 2, and total ILD extent in multivariable model 3. Adjustments were made for age, sex, BMI, and UIP pattern on baseline CT if *p-*values were < 0.2 in univariable analyses. The baseline percent predicted FVC was included, regardless of its significance in univariable analyses, as it reflects the restrictive lung function typical of ILD patients and is associated with prognosis [1, 5, 26]. The C-index of the Cox models was calculated using Harrell’s concordance statistic. Additionally, the added value of incorporating QCT into the FVC analysis was evaluated using the likelihood ratio test to compare nested models with and without QCT information. To more clearly highlight the clinical relevance of QCT, we compared Harrell’s C-index for all-cause mortality between QCT-based changes and visual assessments made by three thoracic radiologists, using CT scans from baseline to the final follow-up during the study period.

For patients with available 1-year follow-up CT scans, logistic regression analyses were conducted to assess changes in each ILD finding, the extent of fibrosis, and the total ILD extent over 1 year. In the Cox proportional hazard regression analyses, we included the variable of 1-year progression, defined as a decline in FVC of ≥ 5% and an increase in fibrosis extent of ≥ 1% over 1 year. The threshold for a 1% increase in fibrosis extent was chosen based on the measurement variability of the software [27].

For sensitivity analyses, we conducted logistic regression using PF-ILD defined exclusively by an absolute decline in FVC of ≥ 10%. Additionally, we included smoking status as an independent variable in patients for whom data were available.

Statistical analyses were conducted using SPSS version 26.0 (IBM Corp.) and R version 4.4.1 (R Core Team, 2024). A *p-*value < 0.05 was considered statistically significant.

## Results

### Baseline and follow-up clinical and radiological characteristics

This study included 465 patients diagnosed with ILD, comprising 238 men with a median age of 65 years (IQR, 58–71 years). Among these patients, 220 (47.3%) were clinically diagnosed with IPF. The remaining 245 patients (52.7%) had non-IPF diagnoses, which included CTD-ILD (*n* = 146 [31.4%]); idiopathic NSIP (*n* = 22 [4.7%]); chronic hypersensitivity pneumonitis (*n* = 5 [1.1%]); cryptogenic organizing pneumonia (*n* = 3 [0.6%]); desquamative interstitial pneumonia (*n* = 1 [0.2%]); and unclassifiable ILD (*n* = 68 [14.6%]).

Baseline CT scans showed that 187 (40.2%) patients exhibited a UIP pattern, 98 (21.1%) displayed a probable UIP pattern, and 180 (38.7%) were indeterminate for UIP or suggested an alternative diagnosis. QCT analysis of the baseline CT scans showed a median of 1.0% (IQR, 0.4–2.4%) GGO, 3.9% (IQR, 1.8–7.2%) RO, 2.0% (IQR, 1.1–3.9%) honeycombing, 6.6% (IQR, 3.3–11.7%) fibrosis extent, and 8.7% (IQR, 4.7–14.9%) total ILD extent. At a median CT follow-up of 44 months (IQR, 33–56 months), these values shifted to 0.9% (IQR, 0.3–3.0%) GGO, 4.9% (IQR, 2.2–9.5%) RO, 2.9% (IQR, 1.3–7.2%) honeycombing, 9.5% (IQR, 4.1–18.3%) fibrosis extent, and 12.1% (IQR, 5.7–21.5%) total ILD extent. Visual assessments by radiologists indicated ILD progression in 264 (56.8%) of the 465 patients. One-year follow-up CT examinations were available for 292 patients (62.8%).

The baseline and follow-up percent predicted FVC values were 81.3 ± 18.1% and 78.2 ± 20.5%, respectively. During the CT follow-up interval, declines in percent predicted FVC of ≥ 5% and ≥ 10% were observed in 177 (38.1%) and 98 (21.1%) of the patients, respectively.

Over the course of the 75-month overall follow-up period (IQR, 59–84 months), 103 (22.2%) patients died (Table 1).

**Table 1:** Baseline clinical and radiological characteristics

Variables	*N* = 465	
Age (years)	65 (58–71)	
Sex (male)	238 (51.2%)	
BMI (< 25 kg/m^2^)	282 (60.6%)	
Smoking status (*n* = 455)		
Never smoker	255 (56.0%)	
Ever smoker	200 (44.0%)	
Clinical diagnosis		
IPF	220 (47.3%)	
Non-IPF	245 (52.7%)	
CTD-ILD	146 (31.4%)	
Idiopathic NSIP	22 (4.7%)	
Chronic hypersensitivity pneumonitis	5 (1.1%)	
Cryptogenic organizing pneumonia	3 (0.6%)	
Desquamative interstitial pneumonia	1 (0.2%)	
Unclassifiable ILD	68 (14.6%)	
ILD pattern on baseline CT		
UIP	187 (40.2%)	
Probable UIP	98 (21.1%)	
Indeterminate for UIP and alternative diagnosis	180 (38.7%)	
CT quantification results	Baseline	24-months follow-up
Ground-glass opacity (%)	1.0 (0.4–2.4)	0.9 (0.3–3.0)
Reticular opacity (%)	3.9 (1.8–7.2)	4.9 (2.2–9.5)
Honeycombing (%)	2.0 (1.1–3.9)	2.9 (1.3–7.2)
Fibrosis extent (%)^a^	6.6 (3.3–11.7)	9.5 (4.1–18.3)
Total ILD extent (%)^b^	8.7 (4.7–14.9)	12.1 (5.7–21.5)
Visual progression on follow-up CT	264 (56.8%)	
CT interval (months)	44 (33–56)	
Pulmonary function test	Baseline	Follow-up
FVC (L)	2.6 (2.1–3.2)	2.4 (1.9–3.1)
% predicted FVC (%)	81.3 ± 18.1	78.2 ± 20.5
Absolute decline in predicted FVC ≥ 5% during follow-up	177 (38.1%)	
Absolute decline in predicted FVC ≥ 10% during follow-up	98 (21.1%)	
Overall follow-up (months)	75 (59–84)	
Death	103 (22.2%)	

Median (interquartile range) is shown for non-parametric variables, and mean ± standard deviation is shown for parametric variables according to the Kolmogorov–Smirnov test

*BMI* body mass index, *IPF* idiopathic pulmonary fibrosis, *CTD-ILD* connective tissue disease-associated interstitial lung disease, *NSIP* nonspecific interstitial pneumonia, *ILD* interstitial lung disease, *FVC* forced vital capacity, *UIP* usual interstitial pneumonia

^a^ Sum of reticular opacity and honeycombing

^b^ Sum of ground-glass opacity, reticular opacity, and honeycombing

### Logistic regression analysis for suggested definitions of PF-ILD

**Fig. 2:**
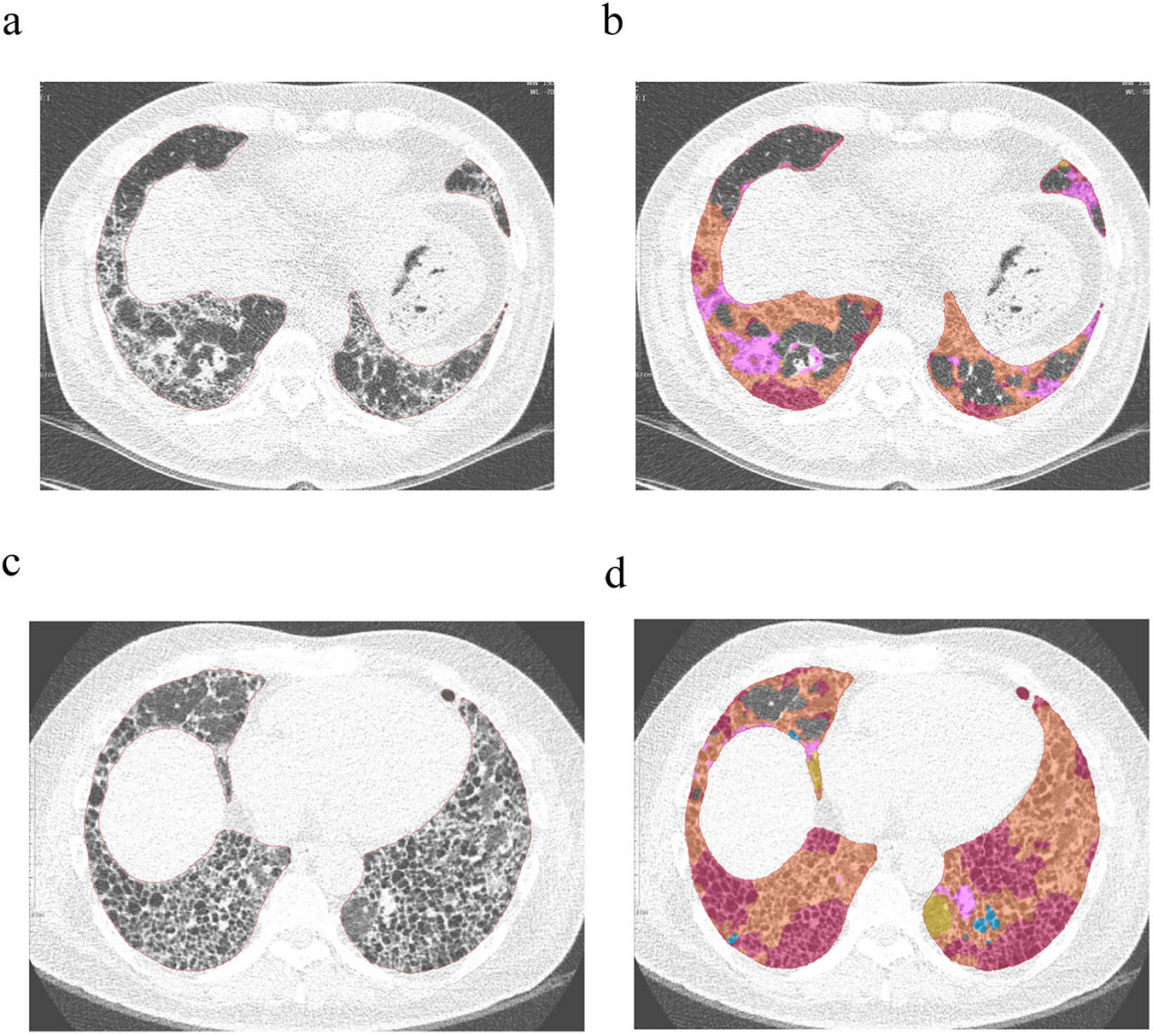
Quantification of interstitial lung disease (ILD) findings on baseline (**a**, **b**) and 29-month follow-up CT (**c**, **d**) of a 57-year-old patient with idiopathic pulmonary fibrosis. The color represents each ILD finding as follows: orange—reticular opacity, red—honeycombing, pink—consolidation, yellow—ground-glass opacity, blue—emphysema. On baseline CT (**a**, **b**), the extent of reticular opacity and honeycombing was quantified as 19.3% and 7.2% of the lung volume, respectively. On follow-up CT (**c**, **d**), the extent of reticular opacity and honeycombing increased to 36.5% and 29.9% of the lung volume, respectively. This case showed a greater extent of reticular opacity on baseline CT than the median 3.9% (interquartile range 1.8–7.2%) of the study population, and was classified as progressive fibrosing interstitial lung disease, with a combination of visual progression of fibrosis on CT and percent predicted FVC decline from 54 to 37%

Among 465 patients, 148 (31.8%) were diagnosed with PF-ILD, characterized by visual CT progression and an absolute decline in percent predicted FVC of at least 5%. Univariable analyses identified several risk factors for PF-ILD: RO extent (odds ratio (OR): 1.076, 95% confidence interval (CI): 1.031, 1.123, *p* = 0.001), fibrosis extent (OR: 1.030, 95% CI: 1.005, 1.056, *p* = 0.020), and total ILD extent (OR: 1.022, 95% CI: 1.000, 1.045, *p* = 0.047) were all significant variables. In multivariable analyses, which were adjusted for clinico-radiological variables, RO extent was confirmed as a risk factor (OR: 1.096, 95% CI: 1.042, 1.152, *p* < 0.001) for PF-ILD. Additionally, fibrosis extent (OR: 1.035, 95% CI: 1.004, 1.067, *p* = 0.025) and total ILD extent (OR: 1.031, 95% CI: 1.006, 1.058, *p* = 0.017) also emerged as significant risk factors in their respective multivariable models (Table 2 and Fig. 2).

**Table 2:** Univariable and multivariable logistic regression analysis for the development of progressive fibrosing interstitial lung disease (ILD) and Cox regression analysis for all-cause mortality

Variables	Development of progressive fibrosing ILD (31.8%, 148 of 465)				All-cause mortality (22.2%, 103 of 465)			
	Univariable analysis		Multivariable analysis		Univariable analysis		Multivariable analysis	
	Odds ratio	*p-*value	Odds ratio	*p-*value	Hazard ratio	*p-*value	Hazard ratio	*p-*value
Ground-glass opacity^a^	0.998 (0.951, 1.047)	0.933			0.936 (0.870, 1.007)	0.078	0.969 (0.906, 1.036)	0.355
Reticular opacity^a^	1.076 (1.031, 1.123)	*0.001*	1.096 (1.042, 1.152)	*< 0.001*	1.100 (1.063, 1.138)	*< 0.001*	1.063 (1.013, 1.115)	*0.013*
Honeycombing^a^	1.013 (0.970, 1.057)	0.564			1.109 (1.078, 1.141)	*< 0.001*	1.074 (1.034, 1.116)	*< 0.001*
Fibrosis extent^b^	1.030 (1.005, 1.056)	*0.020*	1.035 (1.004, 1.067)	*0.025*	1.071 (1.051, 1.091)	*< 0.001*	1.067 (1.043, 1.093)	*< 0.001*
Total ILD extent^c^	1.022 (1.000, 1.045)	*0.047*	1.031 (1.006, 1.058)	*0.017*	1.048 (1.030, 1.066)	*< 0.001*	1.046 (1.026, 1.067)	*< 0.001*

Progressive fibrosing ILD was defined as visual progression on CT and absolute decline in FVC % ≥ 5%

Fibrosis extent was defined as the sum of reticular opacity and honeycombing; total ILD extent was defined as the sum of ground-glass opacity, reticular opacity, and honeycombing; multivariable analyses were adjusted for age, sex, BMI, and UIP on baseline CT, if their *p-*values were < 0.2 in the univariable analyses. Baseline percent predicted FVC was also adjusted, regardless of its significance in the univariable analyses

*ILD* interstitial lung disease, *FVC* forced vital capacity, *UIP* usual interstitial pneumonia, *BMI* body mass index

^a^ Multivariable model 1 was for each ILD finding (ground-glass opacity, reticular opacity, and honeycombing). Ground-glass opacity and honeycombing were not included in the multivariable logistic regression analyses since their *p-*values were > 0.2 in the univariable analyses

^b^ Multivariable model 2 was for fibrosis extent

^c^ Multivariable model 3 was for total ILD extent

The italic values indicate cases where the *p*-value is less than 0.05, signifying statistical significance

The results of logistic regression analyses when PF-ILD was defined by an absolute decline in percent predicted FVC ≥ 10% are described in Supplementary Table 1. Analyses of the subset of patients with available information on their smoking status are described in Supplementary Table 2.

### Cox proportional hazard regression analysis for all-cause mortality

In univariable Cox proportional hazard regression analyses, RO (hazard ratio (HR): 1.100, 95% CI: 1.063, 1.138, *p* < 0.001), honeycombing (HR: 1.109, 95% CI: 1.078, 1.141, *p* < 0.001), extent of fibrosis (HR: 1.071, 95% CI: 1.051, 1.091, *p* < 0.001), and total extent of interstitial lung disease (ILD) (HR: 1.048, 95% CI: 1.030, 1.066, *p* < 0.001) were identified as significant factors for all-cause mortality. In the multivariable analysis, RO (HR: 1.063, 95% CI: 1.013, 1.115, *p* = 0.013) and honeycombing (HR: 1.074, 95% CI: 1.034, 1.116, *p* < 0.001) remained significant poor prognostic factors after adjustment for clinico-radiological variables. Independent multivariable analyses also showed that the extent of fibrosis (HR: 1.067, 95% CI: 1.043, 1.093, *p* < 0.001) and total ILD extent (HR: 1.046, 95% CI: 1.026, 1.067, *p* < 0.001) were significant poor prognostic factors (Table 2). Analyses of patients with available information on their smoking status are described in Supplementary Table 3.

When comparing the C-index, the Cox proportional hazards models that included baseline percent predicted FVC and QCT results (for GGO, RO, and honeycombing, C-index: 0.714, 95% CI: 0.660, 0.764, *p* < 0.001; for fibrosis extent, C-index: 0.703, 95% CI: 0.649, 0.752, *p* < 0.001; for total ILD extent, C-index: 0.666, 95% CI: 0.616, 0.717, *p* < 0.001) demonstrated better predictive accuracy than the model that included only baseline percent predicted FVC (C-index: 0.545, 95% CI: 0.500, 0.599).

For CT scans from baseline to final follow-up during the study period, QCT-based changes in the extent of ground-glass opacity, reticular opacity, and honeycombing (C-index: 0.713; 95% CI: 0.649, 0.771; *p* = 0.003), fibrosis extent (C-index: 0.671; 95% CI: 0.596, 0.733; *p* = 0.023), and total ILD extent (C-index: 0.726; 95% CI: 0.666, 0.778; *p* < 0.001) demonstrated significantly higher prognostic value for all-cause mortality compared to visual assessment of fibrosis progression by radiologists (C-index: 0.604; 95% CI: 0.560, 0.649).

### Subgroup analysis in patients with 1-year follow-up CT images

In univariable logistic regression analyses, baseline RO, an increase in RO during the 1-year follow-up, and an increase in the extent of fibrosis during the same period were all significant risk factors (all ORs > 1, all *p*-values < 0.05). In the multivariable logistic regression analysis, after adjusting for clinico-radiological variables, both the increase in RO during the 1-year follow-up (OR: 1.085, 95% CI: 1.009, 1.167, *p* = 0.027) and the baseline RO on CT (OR: 1.081, 95% CI: 1.012, 1.154, *p* = 0.021) were significant predictors (Table 3).

**Table 3:** Univariable and multivariable logistic regression analysis for development of progressive fibrosing interstitial lung disease (ILD) defined as visual progression of CT and absolute decline in FVC % ≥ 5% in patients with available 1-year follow-up CT images

Model	Variables	Development of progressive fibrosing ILD (32.9%, 96 of 292)			
		Univariable analysis		Multivariable analysis	
		Odds ratio	*p-*value	Odds ratio	*p-*value
Model 1^a^	Baseline ground-glass opacity	0.989 (0.931, 1.051)	0.717		
	Baseline reticular opacity	1.062 (1.007, 1.121)	*0.028*	1.081 (1.012, 1.154)	*0.021*
	Baseline honeycombing	1.011 (0.954, 1.072)	0.705		
	Increase in ground-glass opacity	1.002 (0.950, 1.056)	0.944		
	Increase in reticular opacity	1.081 (1.010, 1.158)	*0.025*	1.085 (1.009, 1.167)	*0.027*
	Increase in honeycombing	1.007 (0.921, 1.101)	0.878		
Model 2^b^	Baseline fibrosis extent	1.025 (0.993, 1.059)	0.125	1.028 (0.987, 1.070)	0.184
	Increase in fibrosis extent	1.069 (1.006, 1.137)	*0.032*	1.060 (0.994, 1.130)	0.074
Model 3^c^	Baseline total ILD extent	1.016 (0.989, 1.044)	0.256	1.027 (0.993, 1.062)	0.116
	Increase in total ILD extent	1.030 (0.990, 1.071)	0.148	1.030 (0.987, 1.075)	0.176

Fibrosis extent was defined as the sum of reticular opacity and honeycombing; total ILD extent was defined as the sum of ground-glass opacity, reticular opacity, and honeycombing; multivariable analyses were adjusted for age, sex, BMI, and UIP on baseline CT, if their *p-*values were < 0.2 in the univariable analyses. Baseline percent predicted FVC was also adjusted, regardless of its significance in the univariable analyses

*ILD* interstitial lung disease, *FVC* forced vital capacity, *UIP* usual interstitial pneumonia, *BMI* body mass index

^a^ Multivariable model 1 was for each ILD finding (ground-glass opacity, reticular opacity, and honeycombing). Baseline and increase in ground-glass opacity and honeycombing were not included in the multivariable analyses since their *p-*values were > 0.2 in the univariable analyses

^b^ Multivariable model 2 was for fibrosis extent

^c^ Multivariable model 3 was for total ILD extent

The italic values indicate cases where the *p*-value is less than 0.05, signifying statistical significance

**Fig. 3:**
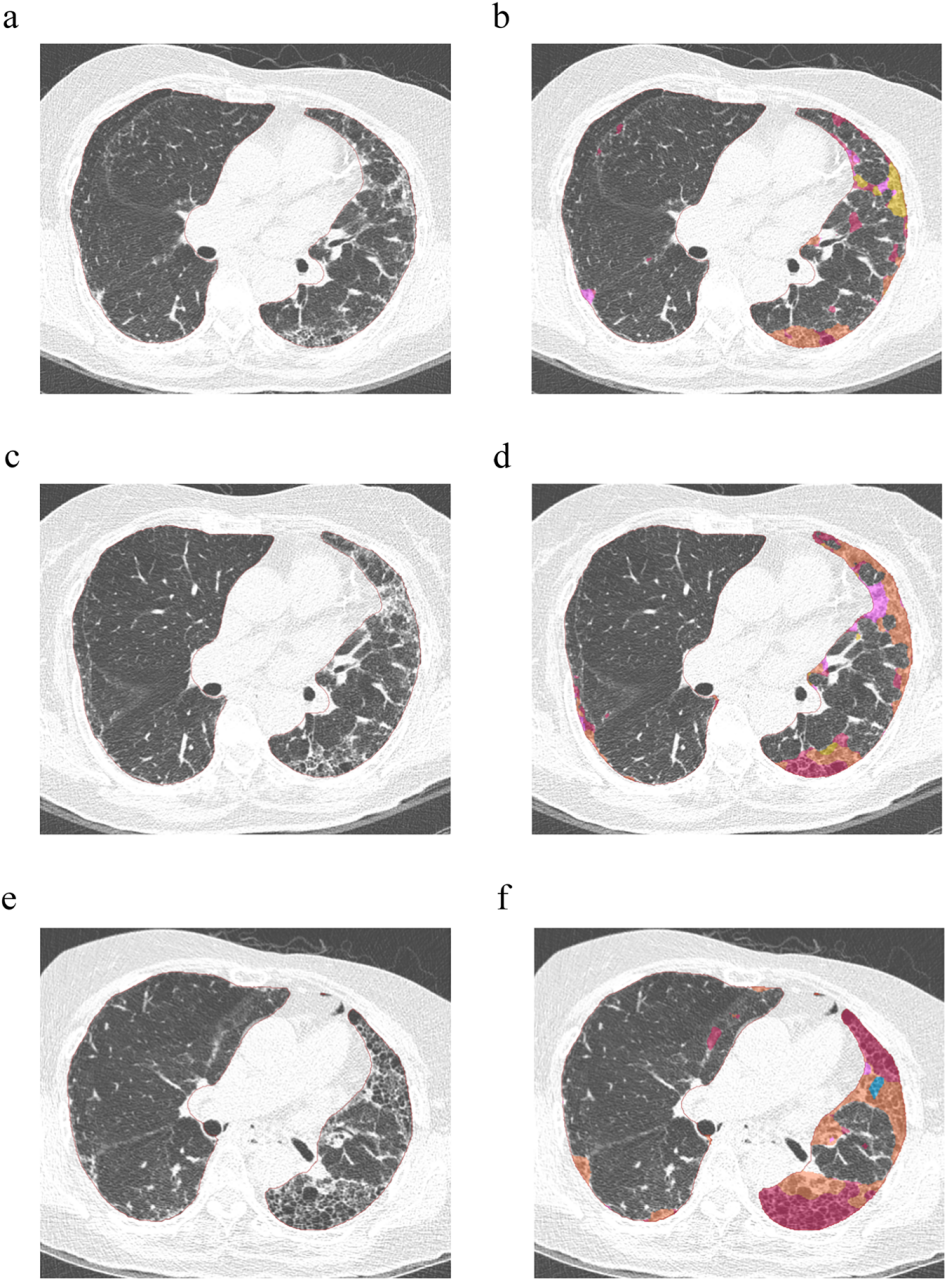
Quantification of interstitial lung disease (ILD) findings on baseline (**a**, **b**), 1-year follow-up (**c**, **d**), and 5-year follow-up CT (**e**, **f**) of a 69-year-old patient with rheumatoid arthritis-associated ILD. The color represents each ILD finding as follows: orange—reticular opacity, red—honeycombing, pink—consolidation, yellow—ground-glass opacity, blue—emphysema. From baseline (**a**, **b**) to 1-year follow-up CT (**c**, **d**), the extent of fibrosis, which is the sum of reticular opacity and honeycombing, increased from 8.6 to 10.7%, and the percent predicted forced vital capacity (FVC) declined from 71 to 65%. This case was defined as progressive fibrosing interstitial lung disease during 1-year follow-up with a combination of more than 1% increase of fibrosis on CT and more than 5% decline in percent predicted FVC. On 5-year follow-up CT (**e**, **f**), the extent of fibrosis further increased to 26.6%, and the patient passed away after 87 months from baseline

In univariable Cox proportional hazard regression analyses, significant poor prognostic factors included baseline reticular opacity, baseline honeycombing, baseline fibrosis extent, baseline total ILD extent, and 1-year progression (all HRs > 1, all *p* < 0.05). In multivariable Cox proportional hazard regression analyses, 1-year progression consistently emerged as a poor prognostic factor across three multivariable models. Specifically, for model 1, the HR was 2.059 (95% CI: 1.095, 3.870; *p* = 0.025); for model 2, the HR was 2.068 (95% CI: 1.105, 3.869; *p* = 0.023); and for model 3, the HR was 2.199 (95% CI: 1.177, 4.109; *p* = 0.014), after adjusting for clinico-radiological variables (Table 4 and Fig. 3).

**Table 4:** Univariable and multivariable Cox regression analysis for all-cause mortality in patients with available 1-year follow-up CT images

Variables	All-cause mortality (20.9%, 61 of 292)			
	Univariable analysis		Multivariable analysis	
	Hazard ratio	*p-*value	Hazard ratio	*p-*value
Baseline ground-glass opacity^a^	0.975 (0.905, 1.051)	0.509		
Baseline reticular opacity^a^	1.089 (1.040, 1.14)	*< 0.001*	1.051 (0.980, 1.127)	0.166
Baseline honeycombing^a^	1.099 (1.055, 1.144)	*< 0.001*	1.044 (0.985, 1.107)	0.149
Baseline fibrosis extent^b^	1.061 (1.035, 1.088)	*< 0.001*	1.047 (1.014, 1.081)	*0.005*
Baseline total ILD extent^c^	1.042 (1.019, 1.066)	*< 0.001*	1.041 (1.013, 1.070)	*0.004*
1-year progression^d^	2.572 (1.393, 4.749)	*0.003*	Model 1^a^	
			2.059 (1.095, 3.870)	*0.025*
			Model 2^b^	
			2.068 (1.105, 3.869)	*0.023*
			Model 3^c^	
			2.199 (1.177, 4.109)	*0.014*

Fibrosis extent was defined as the sum of reticular opacity and honeycombing; total ILD extent was defined as the sum of ground-glass opacity, reticular opacity, and honeycombing; multivariable analyses were adjusted for age, sex, BMI, and UIP on baseline CT, if their *p-*values were < 0.2 in the univariable analyses. Baseline percent predicted FVC was also adjusted, regardless of its significance in the univariable analyses

*PF-ILD* progressive fibrosing interstitial lung disease, *FVC* forced vital capacity, *UIP* usual interstitial pneumonia, *BMI* body mass index

^a^ Multivariable model 1 was for each ILD finding (ground-glass opacity, reticular opacity, and honeycombing). Baseline ground-glass opacity was not included in the multivariable analyses since its *p-*value was > 0.2 in the univariable analyses

^b^ Multivariable model 2 was for fibrosis extent

^c^ Multivariable model 3 was for total ILD extent

^d^ 1-year progression was defined as absolute decline in FVC % ≥ 5% and absolute increase in fibrosis % ≥ 1% over 1 year

The italic values indicate cases where the *p*-value is less than 0.05, signifying statistical significance

Additional logistic regression and Cox proportional hazard regression analyses in the subset of patients with available information on their smoking status are described in Supplementary Tables 4 and 5.

## Discussion

For patients with PF-ILD, initiating standard treatment early—tailored to the specific underlying ILD, such as immunomodulatory therapy for idiopathic NSIP, antigen avoidance for chronic HP, and immunosuppression for CTD-ILD—and using nintedanib as a secondary treatment are associated with improved lung function and patient outcomes [1, 6, 7]. Identifying patients with a high probability of progressive fibrosis enables close monitoring and early intervention. Recent advances in deep learning have facilitated the quantification of CT findings in ILD, allowing for automatic and reproducible disease evaluation. Utilizing these deep learning-based QCT results, we investigated the risk factors for PF-ILD and their outcomes. Consequently, RO, fibrosis extent, and total ILD extent were identified as risk factors for the development of PF-ILD. For all-cause mortality, RO, honeycombing, fibrosis extent, and total ILD extent served as prognostic markers. Incorporating 1-year follow-up CT, both RO and increased RO were predictors of PF-ILD. The 1-year progression based on QCT and FVC demonstrated prognostic value for all-cause mortality.

Deep learning-based QCT has demonstrated the prognostic value of quantitative CT methods in ILD in a prior study. Representatively, Oh et al and Humphries et al employed data-driven texture analysis (DTA) to estimate fibrosis extent on chest CT in cohorts of 979 and 393 patients with ILD and IPF, respectively [30, 31]. In these studies, baseline DTA scores were associated with PFT results, including FVC and DLCO % predicted, as well as with subsequent annual declines in these measures. Furthermore, DTA scores were identified as significant prognostic factors for transplantation-free or progression-free survival, even after adjusting for other clinical variables such as ILD CT patterns and baseline PFT values. Compared to these prior studies [30, 31], we introduced the development of PF-ILD as a separate outcome in addition to all-cause mortality, as patients with PF-ILD had higher mortality than those without [1–4]. By identifying risk factors for PF-ILD, radiologists and clinicians can more proactively monitor and manage high-risk patients. Additionally, we evaluated the prognostic significance of 1-year changes in QCT metrics among patients with ILD. While the extent of fibrosis on baseline CT is important, early changes in fibrosis extent are also key indicators of disease progression [32]. Our study addressed this question by applying a deep learning-based QCT approach.

Although the baseline extent of RO was found to be a significant predictor of PF-ILD, the baseline extent of honeycombing was not. We speculate that since honeycombing is considered to represent end-stage lung fibrosis [33, 34], lung parenchyma with honeycombing has less potential for further fibrosis progression. Conversely, RO may indicate an ongoing process of inflammation and fibrosis. This is supported by recent studies on interstitial lung abnormalities, which have pathologically identified scattered foci of proliferating fibroblasts in areas corresponding to RO on CT scans [35, 36]. Furthermore, we demonstrated that an increase in RO over 1 year was a predictor of PF-ILD, whereas changes in honeycombing were not, reinforcing our hypothesis.

Interestingly, the ILD pattern observed on baseline CT did not significantly predict PF-ILD after adjusting for clinico-radiological variables (Supplementary Table 6). For mortality, the ILD pattern on baseline CT emerged as a significant prognostic factor when adjusted for other clinical factors. However, its significance diminished when baseline QCT results were included in the adjustment (Supplementary Table 7). These findings appear to contradict previous studies that identified the UIP pattern on CT as a risk factor for PF-ILD and a strong prognostic indicator [7, 11, 14, 15]. This discrepancy may stem from the subjective assessment of the UIP pattern on CT. Widell et al reported only fair to moderate interobserver agreement for the UIP pattern on CT, which was lower than that for honeycombing and reticular patterns [37]. Additionally, the retrospective nature of this study means that the baseline CT may not have been performed at the initial phase of the ILD course in some patients. The clinical relevance of the ILD CT pattern may vary depending on when the CT was obtained during the course of ILD. Therefore, further prospective studies with more consistent baseline CT imaging at the onset of ILD are necessary for validation. However, QCT results consistently showed significance (Tables 1, 2), suggesting that QCT assessments of each ILD finding may be clinically more relevant at a random time point than the ILD pattern itself, offering the advantage of objectivity.

In a study conducted by Lee et al, the extent of baseline fibrosis and the interval change in fibrosis extent on QCT were identified as significant predictors of survival in IPF patients [38]. Similarly, our study, which encompassed both IPF and non-IPF ILD patients, demonstrated an association between the extent of fibrosis or its increase and mortality. These findings align with previous research on the prognostic value of fibrosis extent in ILD [2, 15] and underscore the clinical utility of deep learning-based QCT analysis.

This study has several limitations. First, its retrospective design limited the availability of clinical data, particularly regarding symptom aggravation. As a result, the definition of PF-ILD used in this study did not include symptom worsening, which is typically considered a component of PF-ILD definitions [1, 7]. Instead, PF-ILD was defined based solely on pulmonary function decline and radiologic progression on CT. Future prospective studies are warranted to incorporate symptom-based criteria and to assess the prognostic implications of different PF-ILD definitions. In addition, although we demonstrated concordant results in subgroup analyses, 1-year follow-up CT was available in 62.8% (292 of 465 patients), which may introduce bias or unmeasured confounding. Second, the generalizability of our results may be limited, as external validation was not conducted across diverse ILD cohorts or institutions. Therefore, prospective multi-center studies are warranted to confirm the reproducibility and broader applicability of our findings. Third, the use of a single QCT software to quantify ILD findings may limit the generalizability of the results. Lastly, variability in CT machines, protocols, and reconstruction algorithms could affect the consistency and reliability of QCT results.

In conclusion, deep learning-based QCT results for baseline reticular opacity and its changes over 1 year have been identified as risk factors for PF-ILD development in ILD patients. Furthermore, fibrosis quantified from deep learning-based QCT analyses, combined with the definition of 1-year progression using this measure of fibrosis change, has shown prognostic value for all-cause mortality in these patients.

## Supplementary information

ELECTRONIC SUPPLEMENTARY MATERIAL

## Abbreviations

FVC: Forced vital capacity
GGO: Ground-glass opacity
IPF: Idiopathic pulmonary fibrosis
ILD: Interstitial lung disease
PF-ILD: Progressive fibrosing interstitial lung disease
QCT: Quantitative computed tomography
RO: Reticular opacity
UIP: Usual interstitial pneumonia
