# Supplementary material for: Risk factors and prognostic indicators for progressive fibrosing interstitial lung disease: a deep learning-based CT quantification approach
Source: Eur Radiol. 2025 Jun 17;35(12):8151–61. doi: 10.1007/s00330-025-11714-x (PMC12634812; doi:10.1007/s00330-025-11714-x)
Supplement: Supplementary file 1 — ELECTRONIC SUPPLEMENTARY MATERIAL [file 330_2025_11714_MOESM1_ESM.pdf]

Risk Factors and Prognostic Indicators for Progressive Fibrosing Interstitial Lung Disease: A Deep Learning-Based CT Quantification Approach

ELECTRONIC SUPPLEMENTARY MATERIAL

Supplementary Text

CT Image Acquisition

CT examinations were performed using nine types of CT scanners, irrespective of whether intravenous contrast medium was used. The scanners included Brilliance-64, Ingenuity, ICT 256, and Mx 8000 (Philips Medical Systems); Sensation 16 and SOMATOM Definition (Siemens Medical Solutions); Aquilion One (Toshiba); and Discovery CT750 HD and LightSpeed Ultra (GE Medical Systems). All CT images were reconstructed using a sharp kernel with a slice thickness ranging from 1-3 mm, voltage settings of 120-140 kVp, and current settings of 100-200 mAs.

Supplementary Tables

Supplementary Table 1. Univariable and multivariable logistic regression analysis for development of progressive fibrosing interstitial lung disease (ILD) defined with absolute decline in FVC % ≥ 10%.

Variables	Development of progressive fibrosing ILD (21.1%, 98 of 465)			
	Univariable analysis		Multivariable analysis	
	Odds ratio	*p*-value	Odds ratio	*p*-value
Ground-glass opacity [a]	0.970 (0.910, 1.034)	0.346		
Reticular opacity [a]	1.053 (1.005, 1.104)	*0.031*	1.072 (1.015, 1.132)	*0.013*
Honeycombing [a]	1.010 (0.963, 1.060)	0.677		
Fibrosis extent [b]	1.022 (0.994, 1.050)	0.128	1.030 (0.996, 1.065)	0.086
Total ILD extent [c]	1.011 (0.987, 1.036)	0.378	1.017 (0.989, 1.046)	0.235

Note – Fibrosis extent was defined as the sum of reticular opacity and honeycombing; Total ILD extent was defined as the sum of ground-glass opacity, reticular opacity, and honeycombing; Multivariable analyses were adjusted for age, sex, BMI, and UIP on baseline CT, if their *p*-values were < 0.2 in the univariable analyses. Baseline percent predicted FVC was also adjusted, regardless of their significance in the univariable analyses

[a] Multivariable model 1 was for each ILD finding (ground-glass opacity, reticular opacity, and honeycombing). Ground-glass opacity and honeycombing were not included in the multivariable analyses since their *p*-values were > 0.2 in the univariable analyses.

[b] Multivariable model 2 was for fibrosis extent.

[c] Multivariable model 3 was for total ILD extent.

ILD: interstitial lung disease, FVC: forced vital capacity, UIP: usual interstitial pneumonia, BMI: body mass index

Supplementary Table 2. Univariable and multivariable logistic regression analysis for development of progressive fibrosing interstitial lung disease (ILD) defined with visual progression of CT and absolute decline in FVC % ≥ 5% in patients with available smoking status.

Variables	Development of progressive fibrosing ILD (32.3%, 147 of 455)			
	Univariable analysis		Multivariable analysis	
	Odds ratio	*p*-value	Odds ratio	*p*-value
Ground-glass opacity [a]	1.000 (0.953, 1.049)	0.988		
Reticular opacity [a]	1.082 (1.036, 1.131)	*<0.001*	1.101 (1.046, 1.159)	*<0.001*
Honeycombing [a]	1.015 (0.973, 1.060)	0.488		
Fibrosis extent [b]	1.033 (1.007, 1.059)	*0.012*	1.037 (1.006, 1.070)	*0.019*
Total ILD extent [c]	1.025 (1.002, 1.047)	*0.030*	1.033 (1.007, 1.059)	*0.012*

Note – Fibrosis extent was defined as the sum of reticular opacity and honeycombing; Total ILD extent was defined as the sum of ground-glass opacity, reticular opacity, and honeycombing; Multivariable analyses were adjusted for age, sex, smoking status, BMI, and UIP on baseline CT, if their *p*-values were < 0.2 in the univariable analyses. Baseline percent predicted FVC was also adjusted, regardless of their significance in the univariable analyses

[a] Multivariable model 1 was for each ILD finding (ground-glass opacity, reticular opacity, and honeycombing). Ground-glass opacity and honeycombing were not included in the multivariable analyses since their *p*-values were > 0.2 in the univariable analyses.

[b] Multivariable model 2 was for fibrosis extent.

[c] Multivariable model 3 was for total ILD extent.

ILD: interstitial lung disease, FVC: forced vital capacity, UIP: usual interstitial pneumonia, BMI: body mass index

Supplementary Table 3. Univariable and Multivariable Cox regression analysis for all-cause mortality in patients with available smoking status.

Variables	All-cause mortality (22.6%, 103 of 455)			
	Univariable analysis		Multivariable analysis	
	Hazard ratio	*p*-value	Hazard ratio	*p*-value
Ground-glass opacity [a]	0.936 (0.870, 1.008)	0.079	0.967 (0.904, 1.035)	0.338
Reticular opacity [a]	1.104 (1.066, 1.142)	*<0.001*	1.066 (1.015, 1.118)	*0.010*
Honeycombing [a]	1.109 (1.078, 1.141)	*<0.001*	1.073 (1.033, 1.115)	*<0.001*
Fibrosis extent [b]	1.072 (1.053, 1.092)	*<0.001*	1.068 (1.043, 1.093)	*<0.001*
Total ILD extent [c]	1.048 (1.031, 1.066)	*<0.001*	1.046 (1.026, 1.066)	*<0.001*

Note – Fibrosis extent was defined as the sum of reticular opacity and honeycombing; Total ILD extent was defined as the sum of ground-glass opacity, reticular opacity, and honeycombing; Multivariable analyses were adjusted for age, sex, smoking status, BMI, and UIP on baseline CT, if their *p*-values were < 0.2 in the univariable analyses. Baseline percent predicted FVC was also adjusted, regardless of their significance in the univariable analyses

[a] Multivariable model 1 was for each ILD finding (ground-glass opacity, reticular opacity, and honeycombing).

[b] Multivariable model 2 was for fibrosis extent.

[c] Multivariable model 3 was for total ILD extent.

ILD: interstitial lung disease, FVC: forced vital capacity, UIP: usual interstitial pneumonia, BMI: body mass index

Supplementary Table 4. Univariable and multivariable logistic regression analysis for development of progressive fibrosing interstitial lung disease (ILD) defined with visual progression of CT and absolute decline in FVC % ≥ 5% in patients with available smoking status and 1-year follow-up CT images

Model	Variables	Development of progressive fibrosing ILD (33.6%, 95 of 283)			
		Univariable analysis		Multivariable analysis	
		Odds ratio	*p*-value	Odds ratio	*p*-value
Model 1 [a]	Baseline ground-glass opacity	0.992 (0.934, 1.054)	0.796		
	Baseline reticular opacity	1.071 (1.013, 1.132)	*0.015*	1.088 (1.018, 1.163)	*0.013*
	Baseline honeycombing	1.016 (0.958, 1.077)	0.596		
	Increase in ground-glass opacity	1.001 (0.949, 1.055)	0.974		
	Increase in reticular opacity	1.077 (1.006, 1.153)	*0.034*	1.080 (1.005, 1.161)	*0.036*
	Increase in honeycombing	1.011 (0.924, 1.106)	0.808		
Model 2 [b]	Baseline fibrosis extent	1.030 (0.996, 1.064)	0.080	1.031 (0.990, 1.074)	0.136
	Increase in fibrosis extent	1.067 (1.004, 1.134)	*0.038*	1.057 (0.992, 1.127)	0.087
Model 3 [c]	Baseline total ILD extent	1.020 (0.992, 1.048)	0.172	1.030 (0.996, 1.065)	0.081
	Increase in total ILD extent	1.028 (0.988, 1.070)	0.169	1.029 (0.986, 1.073)	0.191

Note – Fibrosis extent was defined as the sum of reticular opacity and honeycombing; Total ILD extent was defined as the sum of ground-glass opacity, reticular opacity, and honeycombing; Multivariable analyses were adjusted for age, sex, smoking status, BMI, and UIP on baseline CT, if their *p*-values were < 0.2 in the univariable analyses. Baseline percent predicted FVC was also adjusted, regardless of their significance in the univariable analyses

[a] Multivariable model 1 was for each ILD finding (ground-glass opacity, reticular opacity, and honeycombing). Baseline and increase in ground-glass opacity and honeycombing were not included in the multivariable analyses since their *p*-values were > 0.2 in the univariable analyses.

[b] Multivariable model 2 was for fibrosis extent.

[c] Multivariable model 3 was for total ILD extent.

ILD: interstitial lung disease, FVC: forced vital capacity, UIP: usual interstitial pneumonia, BMI: body mass index

Supplementary Table 5. Univariable and Multivariable Cox regression analysis for all-cause mortality in patients with available smoking status and 1-year follow-up CT images

Variables	All-cause mortality (32.9%, 96 of 292)			
	Univariable analysis		Multivariable analysis	
	Hazard ratio	*p*-value	Hazard ratio	*p*-value
Baseline ground-glass opacity [a]	0.977 (0.906, 1.052)	0.531		
Baseline reticular opacity [a]	1.095 (1.046, 1.147)	*<0.001*	1.052 (0.979, 1.130)	0.166
Baseline honeycombing [a]	1.100 (1.057, 1.145)	*<0.001*	1.043 (0.984, 1.106)	0.156
Baseline fibrosis extent [b]	1.063 (1.038, 1.089)	*<0.001*	1.047 (1.014, 1.081)	*0.005*
Baseline total ILD extent [c]	1.044 (1.021, 1.067)	*<0.001*	1.041 (1.012, 1.070)	*0.004*
1-year progression [d]	2.596 (1.406, 4.794)	*0.002*	Model 1 [a]	
			2.088 (1.109, 3.930)	*0.023*
			Model 2 [b]	
			2.104 (1.127, 3.927)	*0.020*
			Model 3 [c]	
			2.232 (1.198, 4.160)	*0.011*

Note – Fibrosis extent was defined as the sum of reticular opacity and honeycombing; Total ILD extent was defined as the sum of ground-glass opacity, reticular opacity, and honeycombing; Multivariable analyses were adjusted for age, sex, smoking status, BMI, and UIP on baseline CT, if their *p*-values were < 0.2 in the univariable analyses. Baseline percent predicted FVC was also adjusted, regardless of their significance in the univariable analyses

[a] Multivariable model 1 was for each ILD finding (ground-glass opacity, reticular opacity, and honeycombing). Baseline ground-glass opacity was not included in the multivariable analyses since its P value was > 0.2 in the univariable analyses.

[b] Multivariable model 2 was for fibrosis extent.

[c] Multivariable model 3 was for total ILD extent.

[d] 1-year progression was defined as absolute decline in FVC % ≥ 5% and absolute increase in fibrosis % ≥ 1%

ILD: interstitial lung disease, FVC: forced vital capacity, UIP: usual interstitial pneumonia, BMI: body mass index

Supplementary Table 6. Univariable and multivariable logistic regression analysis for development of progressive fibrosing interstitial lung disease (ILD) defined with visual progression of CT and absolute decline in FVC % ≥ 5%.

Variables		Development of progressive fibrosing ILD (31.8%, 148 of 465)			
		Univariable analysis		Multivariable analysis	
		Odds ratio	*p*-value	Odds ratio	*p*-value
ILD pattern on baseline CT (reference: indeterminate for UIP & alternative diagnosis)	UIP pattern	1.438 (0.917, 2.255)	0.114	Model 1 [a]	
				0.878 (0.534, 1.442)	0.607
				Model 2 [b]	
				0.869 (0.516, 1.464)	0.598
				Model 3 [c]	
				0.970 (0.595, 1.583)	0.904
				Model 4 [d]	
				1.099 (0.683, 1.768)	0.697
	probable UIP pattern	1.792 (1.060, 3.030)	*0.029*	Model 1 [a]	
				1.547 (0.891, 2.684)	0.121
				Model 2 [b]	
				1.550 (0.898, 2.676)	0.115
				Model 3 [c]	
				1.656 (0.956, 2.867)	0.072
				Model 4 [d]	
				1.572 (0.913, 2.708)	0.103

Note – Multivariable analyses were adjusted for age, sex, BMI, and baseline CT quantification results, if their *p*-values were < 0.2 in the univariable analyses. Baseline percent predicted FVC was also adjusted, regardless of their significance in the univariable analyses

Baseline CT quantification results were as follows:

[a] Multivariable model 1 was for each ILD finding (ground-glass opacity, reticular opacity, and honeycombing). Ground-glass opacity and honeycombing were not included in the multivariable analyses since their *p*-values were > 0.2 in the univariable analyses.

[b] Multivariable model 2 was for fibrosis extent.

[c] Multivariable model 3 was for total ILD extent.

[d] Multivariable model 4 was for ILD pattern on baseline CT without adjusting for baseline CT quantification result.

ILD: interstitial lung disease, FVC: forced vital capacity, UIP: usual interstitial pneumonia, BMI: body mass index

Supplementary Table 7. Univariable and Multivariable Cox regression analysis for all-cause mortality

Variables		All-cause mortality (22.2%, 103 of 465)			
		Univariable analysis		Multivariable analysis	
		Hazard ratio	*p*-value	Hazard ratio	*p*-value
ILD pattern on baseline CT (reference: indeterminate for UIP & alternative diagnosis)	UIP pattern	2.568 (1.591, 4.145)	*<0.001*	Model 1 [a]	
				1.019 (0.574, 1.809)	0.948
				Model 2 [b]	
				1.123 (0.650, 1.94)	0.677
				Model 3 [c]	
				1.601 (0.973, 2.634)	0.064
				Model 4 [d]	
				1.924 (1.178, 3.141)	*0.009*
	probable UIP pattern	2.103 (1.186, 3.73)	*0.011*	Model 1 [a]	
				1.772 (0.97, 3.239)	0.063
				Model 2 [b]	
				1.872 (1.035, 3.386)	*0.038*
				Model 3 [c]	
				2.109 (1.164, 3.822)	*0.014*
				Model 4 [d]	
				1.934 (1.071, 3.492)	*0.029*

Note – Multivariable analyses were adjusted for age, sex, BMI, and baseline CT quantification results, if their *p*-values were < 0.2 in the univariable analyses. Baseline percent predicted FVC was also adjusted, regardless of their significance in the univariable analyses

Baseline CT quantification results were as follows:

[a] Multivariable model 1 was for each ILD finding (ground-glass opacity, reticular opacity, and honeycombing).

[b] Multivariable model 2 was for fibrosis extent.

[c] Multivariable model 3 was for total ILD extent.

[d] Multivariable model 4 was for ILD pattern on baseline CT without adjusting for baseline CT quantification result.

ILD: interstitial lung disease, FVC: forced vital capacity, UIP: usual interstitial pneumonia, BMI: body mass index
